# Anomalous wind triggered the largest phytoplankton bloom in the oligotrophic North Pacific Subtropical Gyre

**DOI:** 10.1038/s41598-019-51989-x

**Published:** 2019-10-29

**Authors:** Chun Hoe Chow, Wee Cheah, Jen-Hua Tai, Sin-Fu Liu

**Affiliations:** 1 Department of Marine Environmental Informatics, National Taiwan Ocean University, Keelung, Taiwan (ROC); 2 Institute of Ocean and Earth Sciences, University of Malaya, Kuala Lumpur, Malaysia; 3 Research Center for Environmental Changes, Academia Sinica, Taipei, Taiwan (ROC); 4 Center of Excellence for Ocean Engineering, National Taiwan Ocean University, Keelung, Taiwan (ROC); 5 Center of Excellence for the Oceans, National Taiwan Ocean University, Keelung, Taiwan (ROC)

**Keywords:** Environmental impact, Marine biology, Physical oceanography

## Abstract

In summer 2010, a massive bloom appeared in the middle (16–25°N, 160–200°E) of the North Pacific Subtropical Gyre (NPSG) creating a spectacular oasis in the middle of the largest oceanic desert on Earth. Peaked in June 2010 covering over two million km^2^ in space, this phytoplankton bloom is the largest ever recorded by ocean color satellites in the NPSG over the period from 1997 to 2013. The initiation and mechanisms sustaining the massive bloom were due to atmospheric and oceanic anomalies. Over the north (25–30°N) of the bloom, strong anticyclonic winds warmed sea surface temperature (SST) via Ekman convergence. Subsequently, anomalous westward ocean currents were generated by SST meridional gradients between 19°N and 25°N, producing strong velocity shear that caused large number of mesoscale (100-km in order) cyclonic eddies in the bloom region. The ratio of cyclonic to anticyclonic eddies of 2.7 in summer 2010 is the highest over the 16-year study period. As a result of the large eddy-number differences, eddy-eddy interactions were strong and induced submesoscale (smaller than 100 km) vertical pumping as observed in the *in-situ* ocean profiles. The signature of vertical pumping was also presented in the *in-situ* measurements of chlorophyll and nutrients, which show higher concentrations in 2010 than other years.

## Introduction

Ocean eddies are abundant in the North Pacific Subtropical Gyre (NPSG)^1–3^. They typically move westward at a speed of about 0.1 m/s^2,4,5^, forming high spatial variability in sea surface height (SSH) which is similar to that of linear Rossby waves^4^. The nonlinear ocean eddies are stronger in spring and summer^1^ and in years when background sea surface temperature (SST) front is strong^3^. During the strong SST front periods, vertical shear between the eastward-flowing Subtropical Countercurrent (STCC) and the westward-flowing North Equatorial Current (NEC) increased, inducing baroclinic instability that is the essential ingredient for eddies to grow^3^. The eddy variability associated with the Hawaii Lee Countercurrent (HLCC) is also determined by the vertical-shear-induced baroclinic instability on the seasonal^6^ and interannual-to-decadal^7^ time scales.

Eddies play a significant role in influencing the marine environments in the NPSG. The eddy-induced meridional heat transport on subsurface can exist along the subtropical SST fronts^8^. The “cusp-shaped” SST fronts induced by the ocean eddies can be found during spring, forming warm and cold SST tongues^5^.

Chlorophyll (CHL) concentrations are generally low in the NPSG because of nutrient limitation^9^. Seasonally, the CHL concentration is the lowest in summer with a climatological value smaller than 0.06 mg/m^3^ due mainly to a constrained in mixing of essential nutrients to the sunlit layer^10^ and phytoplankton lowering the production of CHL in response to high light in the shallower mixed layer^11,12^. Although deprived of nutrients in summer, phytoplankton blooms have been reported in the NPSG with most of the blooms located in the eastern part of the NPSG and closed to the Hawaiian Islands^13–17^. Interestingly, most of these blooms occur almost annually^14^. In contrast, blooms are rarely reported in the western NPSG with the exception of a large bloom (about 2000-km long) reported in 2003^18^.

**Figure 1:**
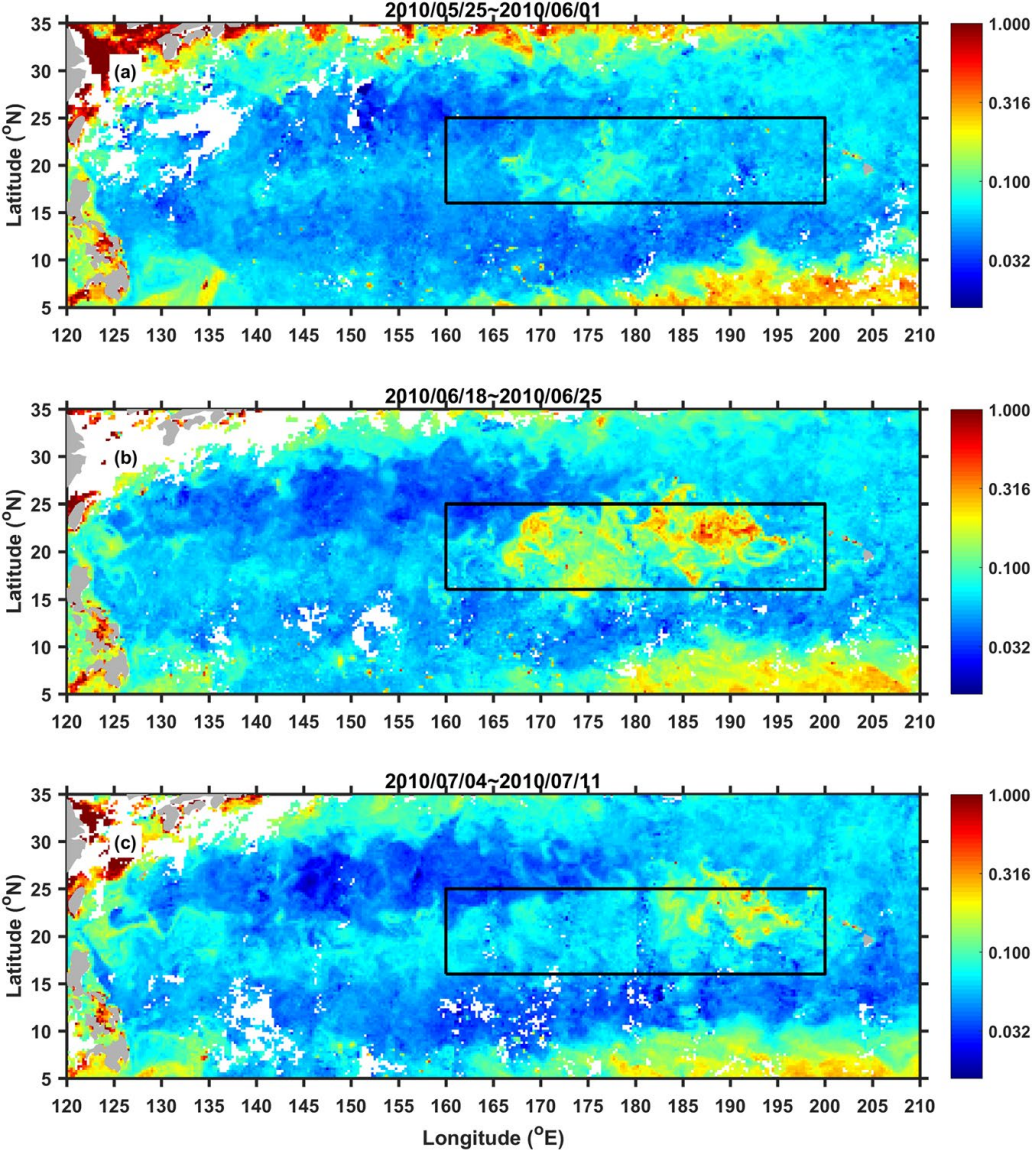
Eight-day averaged satellite CHL concentration (unit in mg/m^3^, log_10_ scale) from (**a**) May 25 to June 1, (**b**) June 18 to June 25 and (**c**) July 4 to July 11, 2010. Black box in each sub-figure shows the region of interest within 160–200°E and 16–25°N. This figure was generated using MATLAB (version R2017a https://www.mathworks.com/).

In this study, we report the largest phytoplankton bloom ever observed by ocean color sensors in the NPSG. The bloom occurred in summer 2010 and appeared as a long zonal band along 20°N between Taiwan and Hawaii. Higher CHL concentrations resided within the 160°E and 200°E band (Fig. 1). Using a combination of satellite remote sensing and *in-situ* measurements, we showed that the eastward propagating bloom was directly related to eddies which induced vertical pumping as a result of anomalous wind-triggered westward currents.

## Results

### The largest summer bloom in the central NPSG from 1998 to 2013

Located in between Taiwan and Hawaii (Fig. 1a), the bloom started as a long zonal band feature with high CHL concentration waters emerging roughly within 15–25°N and 165–185°E. Chronologically, the bloom first occurred at the end of May and propagated eastward. By the third week of June, the bloom has reached its peak covering almost 40° in longitude. After that, the bloom began to decline but still propagating further east to about 200°E closer to Hawaii in July 2010 (Fig. 1c). CHL concentration within the bloom ranged from 0.1 to 0.3 mg/m^3^ (Fig. 1a). At its peak, the CHL concentration can reach as high as 1 mg/m^3^ (Fig. 1b). Even when the CHL concentration decreased, the zonal band feature of the bloom can still be seen clearly in between Taiwan and Hawaii (Fig. 1c).

**Figure 2:**
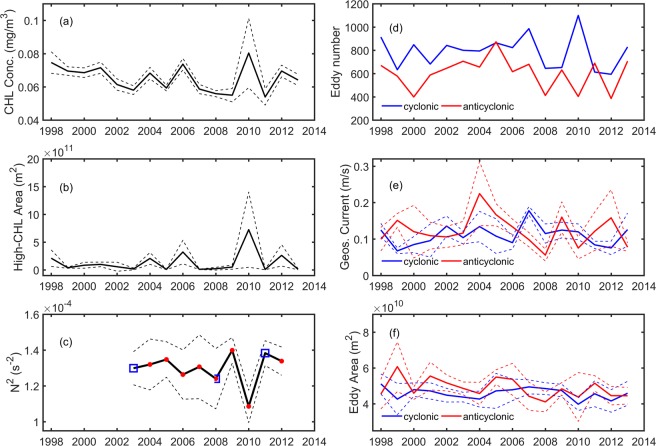
Interannual variability of CHL and physical parameters averaged within the 160–200°E and 16–25°N region (see the black box in Fig. 1 for the region) obtained in June and July. (**a**) Mean CHL concentration. (**b**) Mean area with CHL concentration >0.1 mg/m^3^. (**c**) CTD-obtained buoyancy frequency averaged over 200 m from the JPBN and JGQH cruises along the 165°E transect. (**d**) Total eddy number. (**e**) Mean geostrophic-current speed of eddies. (**f**) Mean eddy area. Red dots and blue squares in (**c**) represent the CTD observation executed in June and July, respectively (the time series without points show no available CTD observation in June or July). Blue and red curves in the right panels are for cyclonic and anticyclonic eddies, respectively. All dashed curves show the plus-minus standard deviations of the mean analysis for the relative parameters.

**Figure 3:**
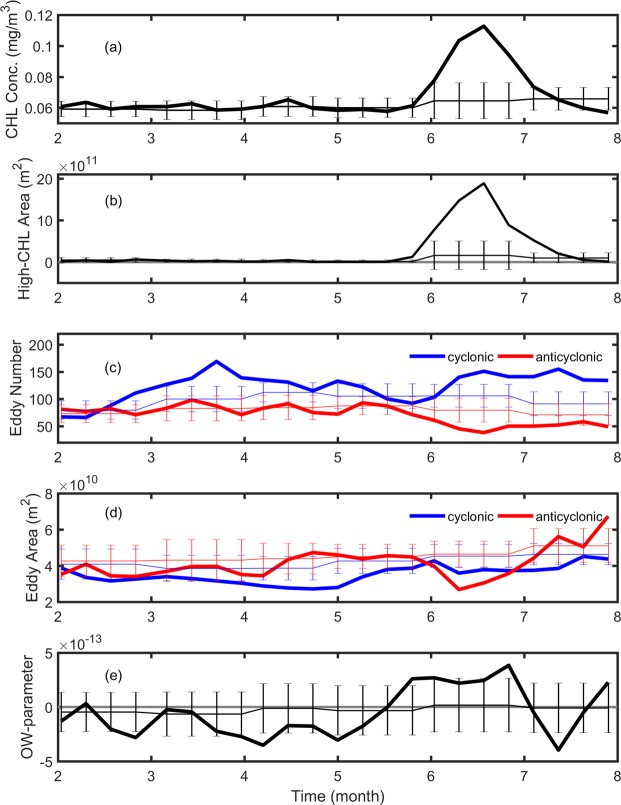
Eight-daily time series of (**a**) CHL concentration, (**b**) area with CHL concentration >0.1 mg/m^3^, (**c**) eddy number, (**d**) eddy area and (**e**) Okubo-Weiss parameter, within 160–200°E and 16–25°N from February to July, 2010. The thin curves with error bars show the climatological monthly mean with the plus-minus standard deviations.

Considering only the region of interest (i.e. black box in Fig. 1) and only for the month of June and July, the mean CHL concentration (about 0.08 mg/m^3^) observed in 2010 is the highest in the period from 1998 to 2013 (Fig. 2a). Also, when considering only the area with CHL concentrations higher than 0.1 mg/m^3^, the bloom’s area is the largest in 2010 reaching around two million km^2^. On the month-to-month time scale from May to July of 2010, Fig. 3a,b show the 8-day variability of CHL concentration and of high-CHL-concentration area averaged over the region of interest, respectively. The bloom first occurred at the end of May, peaked at mid-June and vanished by the end of July, 2010. From Fig. 3a,b, it is clear that CHL concentrations and high-CHL-concentration area are significantly higher than the climatological mean from 1998 to 2013.

### The anomalies of oceanic fields and winds in 2010

In the NPSG, anticyclonic eddies are generally more abundant than cyclonic eddies in total number in the STCC zonal band from 122°E to 170°E between 12°N and 28°N, except between 19°N and 22°N^19^. In summer, however, the number of cyclonic eddies are generally more than the anticyclonic eddies (Fig. 2d) within the bloom region (160–200°E and 16–25°N). Particularly in 2010, the number of cyclonic eddies were 2.7 times more than the anticyclonic eddies (Fig. 2d), which is the highest from 1998 to 2013, showing the dominance of cyclonic eddies. Moreover, the cyclonic and anticyclonic eddies have similar geostrophic-current speeds (Fig. 2e) and areas (Fig. 2f) in 2010. On the month-to-month time scale, Fig. 3c shows an increasing in the cyclonic-eddy number and a decreasing in the anticyclonic-eddy number in June 2010. The huge differences in the cyclonic-anticyclonic eddy numbers in 2010 are significant in comparison to the climatology of eddy numbers (Fig. 3c). In addition, the size of the cyclonic and anticyclonic eddies was also anomalously smaller during the bloom-peak period in June when comparing to the climatological eddy fields (Fig. 3d). The coincidence of eddy size and eddy number with the bloom occurrence suggests a bloom mechanism related to the ocean eddies in summer 2010.

**Figure 4:**
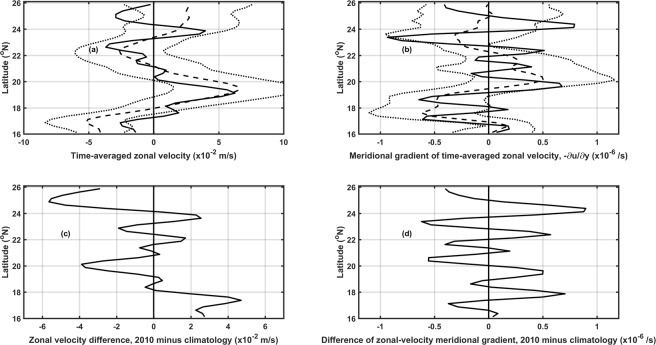
Meridional distribution of zonal (**a**) velocity and (**b**) velocity shear, within 160°E and 200°E, in June and July, averaged from 1998 to 2013 (dashed) and averaged in 2010 (solid). (**c**,**d**) Differences between the 2010 and climatological fields. The dot curves in (**a**,**b**) show the standard deviation for the climatological fields.

Since eddy variability is mainly determined by the changes of STCC^1,3^ and HLCC^6,7^, the background currents were examined to study the possible mechanism for the eddy-number difference in 2010. Figure 4 shows the background zonal currents represented by the satellite-obtained absolute geostrophic currents and relative velocity horizontal shear in the region of interest during June and July of 2010, comparing to the climatological fields from 1998 to 2013. The STCC and HLCC can be seen in Fig. 4a (dashed curve), centered along 24–25°N and 19–20°N^20^, respectively, flowing eastward at a speed reaching 2.5–5 cm/s. Between these subtropical countercurrents, a westward current flows at a maximum speed of about 2.5 cm/s, centered along 22°N. In Fig. 4b (dashed curve), current velocity shear is mostly positive except within the 20°N and 22°N region.

In contrast to the climatological fields, anomalous westward currents are found at 25°N (Fig. 4a,c) in 2010, inducing anomalous changes in current velocity shear (Fig. 4b,d) that could change potential vorticity and cause eddy polarity-asymmetry (more cyclonic eddies and less anticyclonic eddies) to the south. According to the quasi-geostrophic theory^21^, there is a necessary condition for barotropic instability or baroclinic instability with a shear flow, when the dominant sign change is mainly due to horizontal shear or vertical shear. Thus, the shear instability induced by the anomalous westward currents could contribute to the eddy polarity-asymmetry during the bloom period.

**Figure 5:**
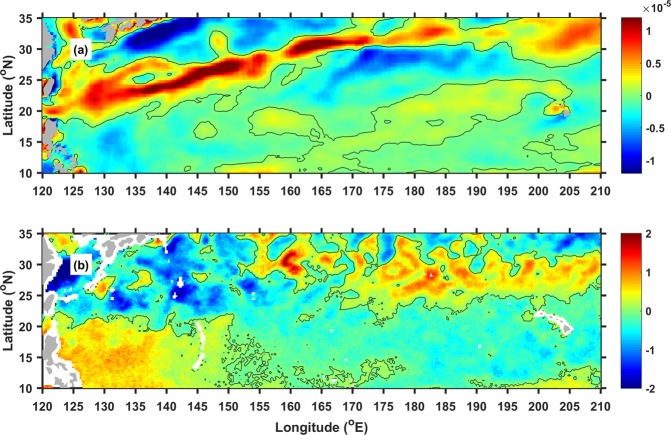
Spatial distribution of (**a**) wind-curl anomalies (s^−1^) and (**b**) SST anomalies (°C) in June and July, 2010. This figure was generated using MATLAB (version R2017a https://www.mathworks.com/).

Oceanic background flow is determined by surface winds over the NPSG^3,20,22^. In spring, strong cyclonic wind anomalies in the north of the eastward-flowing STCC enhanced the SST front by decreasing SST via Ekman suction^22^. Anomaly fields of anticyclonic winds and warm SST are found roughly within 160–200°E and 25–30°N, north of the bloom and westward flows (Fig. 5). The strong anticyclonic winds warm the SST beneath via Ekman convergence. Then, positive meridional SST gradients are formed south of the strong anticyclonic winds, strengthening the westward flows in the STCC region according to the thermal wind balance^3,20,22^.

### Ocean vertical profiles in 2010

Large number difference in cyclonic and anticyclonic eddies can generate strong eddy-eddy interactions. The strength of eddy-eddy interactions was derived using the Okubo-Weiss (OW) parameter^23–25^, which can provide the difference between deformation rate and vorticity. The OW-parameter averaged within the region of interest in summer 2010 is about 1 × 10^−13^ s^−2^, showing stronger eddy deformation than eddy rotation. The OW-parameter switched from negative to positive values at the end of May, and then was in positive values in June (Fig. 3e). This shows that the eddy deformation rate is larger than the eddy vorticity during the bloom period, suggesting strong deformation fields among the eddies. Under these mesoscale circumstances, submesoscale vertical pumping can be induced in the high-velocity regions surrounding both types of eddies^26^, via horizontal stretching done by eddy deformation fields during eddy-eddy interactions^27^.

**Figure 6:**
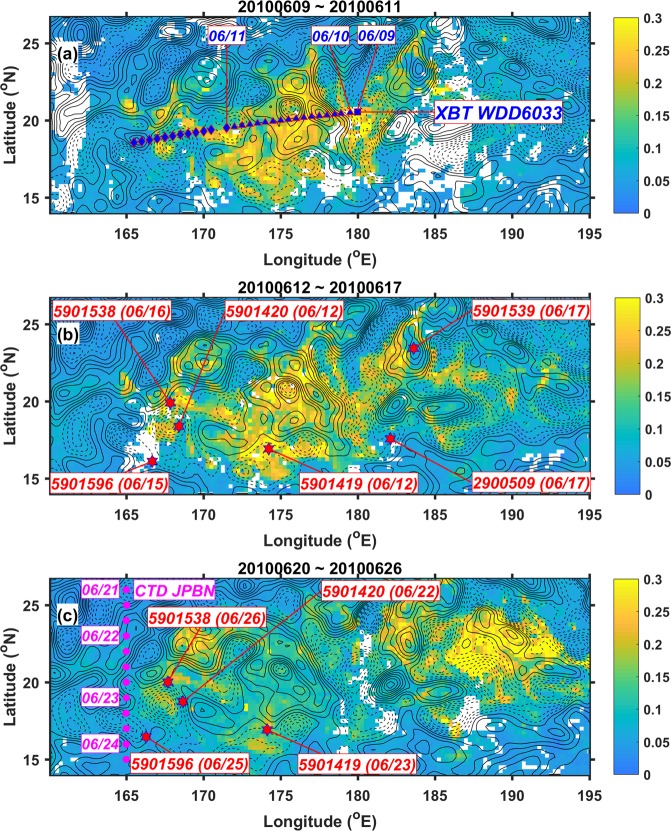
Distribution of CHL concentration (color shaded, units: mg/m^3^) and SSHAs (contours with 2.5-cm interval) averaged from (**a**) June 9 to June 11, (**b**) June 12 to June 17 and (**c**) June 20 to June 26, covering the measurement periods of WDD-6033 XBT, Argo floats and JPBN CTD. Dashed and solid contours are for the negative and zero-positive SSH anomalies, respectively. The square, triangles and diamonds marked in blue in (**a**) show the locations of WDD-6033 XBT on June 9, 10 and 11, respectively (the locations pointed to the dates show the first XBT locations on the particular dates). The red stars in (**b**,**c**) show the Argo-float locations, pointed to the noted platform numbers that are followed by the dates in the parenthesis. The purple circles show the locations of CTD from June 21 to 24 (the locations with dates at their left sides show the first CTD locations on the particular dates). This figure was generated using MATLAB (version R2017a https://www.mathworks.com/).

To study the relevant ocean vertical structure, we used publicly available hydrographic profiles observed by conductivity-temperature-depth devices (CTD), Argo buoys and expendable bathythermographs (XBT) that passed through the bloom region. Locations and platform numbers/names of the profiles were superimposed on the maps of CHL concentrations and sea-surface-height anomalies (SSHAs) from June 9 to June 26 (Fig. 6), a duration that covers the whole observation periods of CTD, Argo buoys and XBT. Locations of the profiles corresponding to the regions with high CHL concentrations and eddy cores can be observed in Fig. 6. An XBT transect passed through the bloom area at about 18–20°N (Fig. 6a) during the growing phase of the bloom (Fig. 3a), whereas a meridional CTD transect at 165°E was available during the decline phase of the bloom (Fig. 6c).

Two Argo buoys with platform numbers of 5901596 (the most southwest red dot in Fig. 6) and 2900509 (the most southeast red dot in Fig. 6) observed the background vertical structures corresponding to low CHL concentrations ( < 0.05 mg/m^3^) observed by satellites, but the others with platform numbers of 5901538, 5901420, 5901419 and 5901539 were in the bloom region with CHL concentrations higher than >0.1 mg/m^3^.

**Figure 7:**
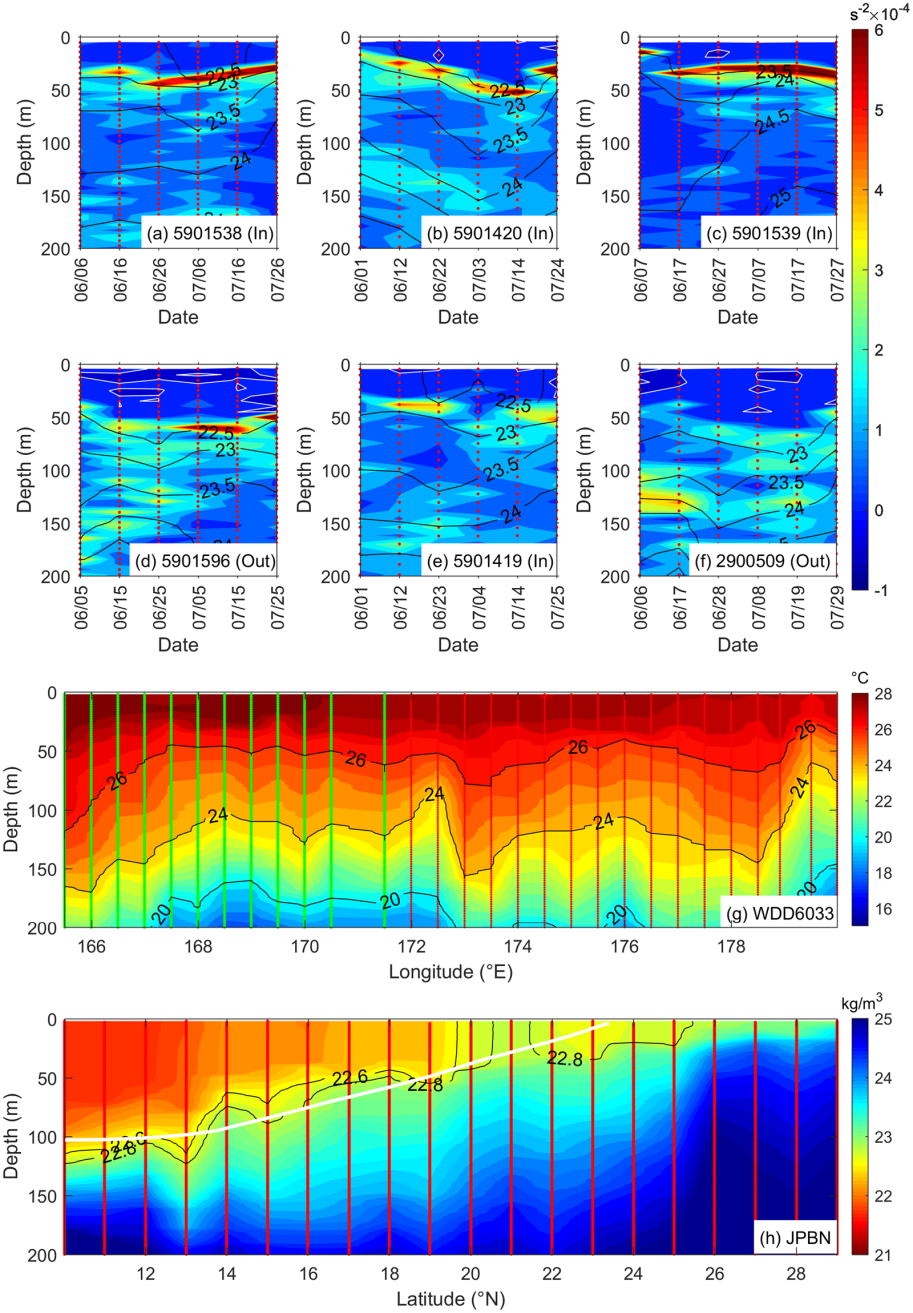
Ocean vertical structures observed from different instruments passing through the bloom region in summer 2010. (**a**∼**f**) Buoyancy frequencies (colors) and potential density (contours, sigma-theta in kg/m^3^) obtained from Argo buoys with the platform numbers of 5901538, 5901420, 5901539, 5901596, 5901419 and 2900509 from June to July (“In” and “Out” in the parenthesis noted after the platform numbers indicate the Argo buoys inside and outside the bloom region, respectively). (**g**) *In-situ* ocean temperature profiles observed by XBT on the WDD6033 platform executed from east to west during June 9 to 11. (**h**) Potential density (sigma-theta in kg/m^3^) obtained from CTD on the JPBN platform executed from north to south during June 20 to 26 (colors and black contours). The white contour shows the climatology sigma-theta 22.8-kg/m^3^.

Inside the bloom region, Fig. 7a–c,e show that the buoyancy frequencies were lower below 50 m and the mixed layer depth is shallower than outside the bloom region shown in Fig. 7d,f. Moreover, the mixed layer stratification is weaker during the bloom growing period in the first half of June than during the declining period after middle of June. These comparisons to the background fields show that the bloom occurrence was under an ocean condition that the mixed-layer stratification was weak and shallow, favorable for nutrient uplifting to the sunlit layer. Furthermore, Fig. 2c shows the interannual variability of buoyancy frequencies based on the CTD transect data obtained from the JPBN and JGQH platforms executed along 165°E from 2003 to 2012. The buoyancy frequencies reached as low as 1.1 × 10^−4^ s^−2^ in 2010, the lowest among the years. The weakest vertical stratification in summer 2010 shows that the ocean provides the best ocean condition for the nutrient uplifting.

**Figure 8:**
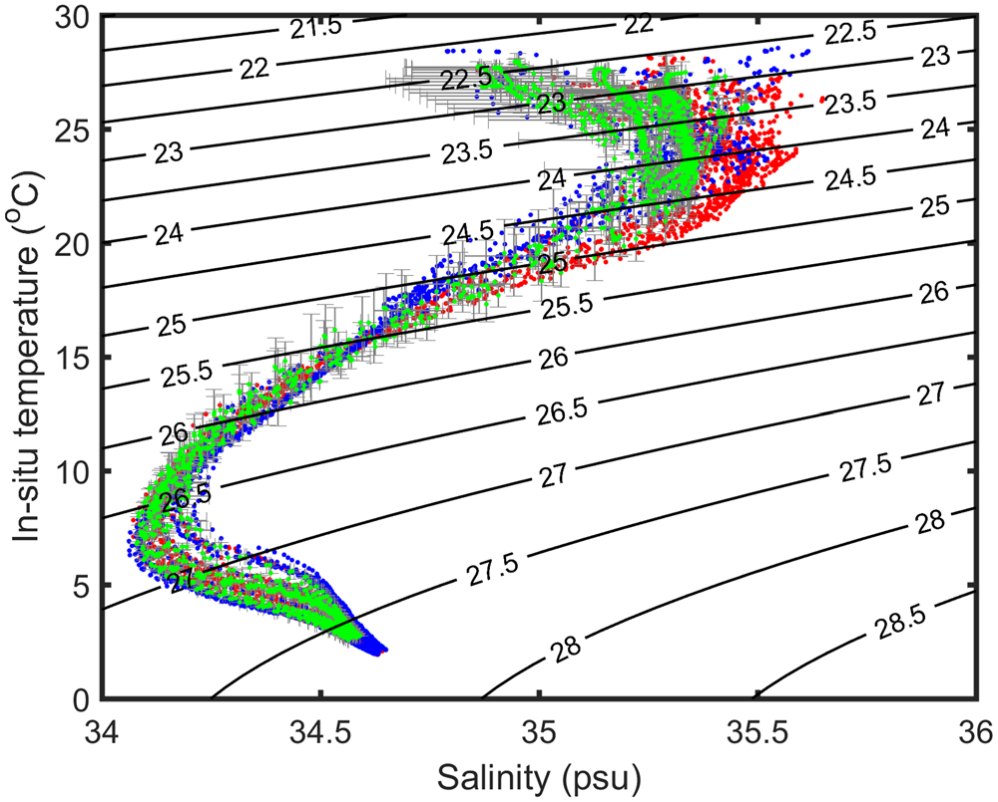
Temperature-salinity (TS) diagram with density sigma-t (contours) based on Argo floats in the region of interest in June and July. Red dots are for measurements with CHL concentration larger than 0.1 mg/m^3^ at the surface in 2010. Blues dots are for those with CHL concentration smaller than 0.05 mg/m^3^ at the surface in 2010. Green dots are for those climatological TS corresponding to red dots. Horizontal and vertical error bars in gray show the standard deviation for the climatological salinity and temperature, respectively.

Water properties with high (>0.1 mg/m^3^) and low (<0.05 mg/m^3^) CHL concentrations are compared (Fig. 8) using all Argo-buoy data available in the region of interest during the bloom period. The temperature-salinity (TS) diagram in Fig. 8 shows that the water properties corresponding to the high and low CHL concentrations are significantly different. The TS properties where the satellite-observed CHL concentrations are high (>0.1 mg/m^3^) were colder and saltier than the waters with lower CHL concentrations (<0.05 mg/m^3^) and climatology. Statistically over 50 m (roughly the mixed-layer depth), the temperature in the high-CHL regions is about 0.57 °C colder than that in the low-CHL regions; while the density and salinity in the high-CHL regions is about 0.28 kg/m^3^ and 0.13 psu larger than those in low-CHL regions, respectively. These comparisons suggest the results of vertical pumping by bringing up colder and denser waters to the mixed layer from below.

Figure 7g shows the temperature profiles observed by the XBT on the WDD6033 platform passing through the bloom region from east to west during June 9 to 11 (see Fig. 6a for corresponding locations of XBT stations). Shown by the concave upward 24°C isotherm, vertical pumping/upwelling can be observed at 172.5°E, 176°E and 179.5°E. The depth of 24°C waters can reach as shallow as 80 m at 172.5°E and 179.5°E, having a horizontal length scale of submesoscale (smaller than 100 km), located at where the eddy currents are strong near boundaries between the pairs of mesoscale cyclonic and anticyclonic eddies (see Fig. 6a for corresponding locations). The depths of isotherm at 26°C, 24°C and 20°C are shallower at 176°E than the vicinity, located near the boundary of a mesoscale cyclonic eddy (see Fig. 6a for corresponding locations).

Moreover, Fig. 7h shows the potential density profiles observed by the CTD on the JPBN platform passing through the west boundary of the bloom region from north to south during June 20 to 26. The potential density (sigma-theta) contours of 22.6 kg/m^3^ and 22.8 kg/m^3^ show the spatial horizontal variability of thermocline from 10°N to 26°N. The outcropping thermocline and denser (>22.8 kg/m^3^) waters can be observed within 20°N and 22°N (see Fig. 6c for corresponding locations), indicating vertical upward motion where the eddy current speed was fast.

**Figure 9:**
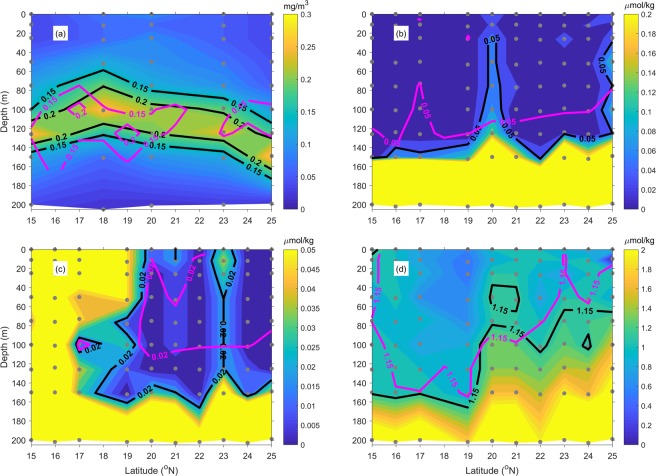
Vertical profiles of (**a**) CHL, (**b**) nitrate + nitrite, (**c**) phosphate and (**d**) silicate along the 165°E transect of JPBN CTD in June 2010. The purple contours show the climatology monthly mean of June according to the CTD data available from 2003 to 2012. The gray dots show the data points of profiles for plotting the contours.

Figure 9 shows the concentration profiles of CHL, nitrate + nitrite, phosphate and silicate along the 165°E transect in June 2010 and climatology fields from 2003 to 2012. The deep CHL maximum layer was at about 100–150 m in 2010. Higher CHL concentrations can be found at the 18° and 20°N stations when compared to the climatology profile. Within 20°N and 21°N, higher concentration of nutrients can be observed (Fig. 9) at where the isotherm outcropped (Fig. 7h). Noting that, Figs 3a and 6c show the nutrient sampling nearly outside the bloom region during the decline of bloom. Thus, the nutrient concentration is not remarkably high as shown in Fig. 9, but the occurrence of upwelling still can be seen in the vertical distribution of nutrients.

## Summary

In this study, we reported an enormous phytoplankton bloom in the eddy-abundant NPSG during summer 2010. The bloom is the largest in area ever recorded by ocean color satellites in the NPSG over the period from 1997 to 2013, determined by the top-down processes of atmosphere and ocean from large scale to submesoscale.

Started from the large-scale process of air-sea interaction north of the bloom, strong anticyclonic winds warmed SST subsequently causing positive meridional SST gradients and inducing anomalous westward flows of ocean surface in the NPSG. Ocean mesoscale variability (100 km in order) responded to the velocity shear of the anomalous westward flows, by increasing the number of cyclonic eddies. This resulted in higher ratio of cyclonic to anticyclonic eddies. Subsequently, eddy deformation became more dominant than eddy rotation during the bloom period, causing strong eddy-eddy interactions. As a consequence of the strong eddy-eddy interactions submesoscale vertical upwelling (smaller than 100 km) was generated, bringing up nutrients below the sunlit layer to euphotic depth.

The submesoscale vertical upwelling and relevant ocean vertical structures favorable for nutrient uplifting were observed using available historical *in-situ* ocean profile observation obtained from Argo buoys, XBT and CTD sampling that passed through the bloom. The ocean stratification above 200 m was the weakest in summer 2010, by comparing to other years. At the beginning of the bloom occurrence, the mixed layer was shallower with weaker stratification inside the bloom region than outside. Above the mixed layer depth, colder and denser waters were observed at where the CHL concentrations were high during the bloom period. The submesoscale vertical upwelling was observed where eddy flows were fast near eddy boundaries. The corresponding higher nutrients and CHL observed from *in-situ* measurements show that a combination of atmospheric and oceanic anomalies is responsible for the largest bloom in the NPSG.

## Data and Methods

We used 8-day CHL data at 1/4° resolution, available since 1997, obtained from the European Space Agency’s GlobColour project (http://www.globcolour.info), which provides a merged CHL product with better coverage from sensors such as Sea-Viewing Wide Field-of-View Sensor (SeaWiFS), Medium Resolution Imaging Spectrometer (MERIS), Moderate Resolution Imaging Spectroradiometer (MODIS) onboard Aqua satellite, and Visible Infrared Imaging Radiometer Suite (VIIRS). We applied the daily altimetry SSHAs and absolute dynamic topographic (ADT) at 1/4° resolution obtained from the Archiving, Validation, and Interpretation of Satellite Oceanographic (AVISO) data (http://www.aviso.altimetry.fr), to study the changes of SSHAs and background flows. Provided by *Faghmous et al*.^25^, the “daily global mesoscale ocean eddy dataset” from satellite altimetry was used to analyze eddy variability, available from 1993 to 2013.

For data consistency, the daily data of SSHAs, ADT and eddies were averaged at 8-day intervals according to the dates of CHL data. Moreover, we analyzed the data from 1998 to 2013 that covered the duration of eddy dataset and CHL data. The geostrophic velocity anomalies (u′, v′) and total geostrophic velocities (u, v) were then derived from the zonal and meridional gradients of the 8-day SSHAs and ADT^2^, respectively. The eddy variability was studied based on the given data of eddy numbers, eddy area, eddy geostrophic-current speed. Only the eddies with amplitude larger than 4 cm were considered due to the SSH accuracy of satellite altimeter of about 4 cm in the open ocean^28^. *Faghmous et al*.^25^ defined eddies as the outermost closed-contour sea level anomalies (SLA) containing a single extreme (maximum/minimum). Bounded by the outermost closed-contour SLA, the eddy area was estimated as the surface area and the eddy geostrophic-current speed was the geostrophic-current speed averaged in the eddies. There were two uncertainties associated with the eddy-identification algorithm of *Faghmous et al*.^25^: (1) the eddy boundary was not necessarily associated with the eddy physical properties, and (2) small features might not be detected. However, the eddy-identification algorithm recovered about 96% of eddy features as identified by domain experts^25^.

To study eddy-eddy interactions, the Okubo-Weiss (OW) parameter^23–25^ was calculated using

1 $$OW={{S}_{sh}}^{2}+{{S}_{st}}^{2}-{\xi }^{2}\ldots $$

where *S*_*sh*_ is the shear-deformation rate estimated as ∂v′/∂x + ∂u′/∂y, *S*_*st*_ is the stretch-deformation rate estimated as ∂u′/∂x − ∂v′/∂y and *ξ* is the vorticity estimated as ∂v′/∂x − ∂u′/∂y, using the geostrophic velocity anomalies (u′, v′). If the eddy-eddy interactions are strong, one particular circular eddy can be stretched by the deformation field of the nearby eddies^27,29^, causing the deformation rate (*S*_*sh*_^2^ + *S*_*st*_^2^) larger than the vorticity (*ξ*^2^) and thus getting positive OW-parameter. Conversely, when the eddy-eddy interactions are weak, the stretched eddy recovers to a circular shape by increasing its vorticity. Thus, the deformation rate becomes smaller than the vorticity, having the negative OW-parameter.

To observe ocean vertical structure during the bloom occurrence, we applied the ocean-profile data of CTD, XBT and Argo buoys. These *in-situ* ocean-profile data were collected and made available by the “Coriolis project and programmes” (http://www.coriolis.eu.org). Only those Argo-buoy data with good-flagged quality were applied. For the bio-chemical parameters, we analyzed the publicly available concentration profiles of CHL, nitrate + nitrite, phosphate and silicate measured along the 165°E transect by the Japan Meteorological Agency (JMA). CHL were measured fluorometrically using a Turner Designs 10-AU-005-CE Field Fluorometer and nutrients were analyzed by Auto Analyzer III (BLTEC, Japan). The precision for nitrate and phosphate expressed as a coefficient of variation (CV), were between 0.2−0.4%^30^.

Moreover, to study the ocean background forcing, we used the monthly reanalysis 10-m winds and satellite-derived SST on 0.25° grids. The 10-m monthly winds data were averaged from daily data obtained from the European Centre for Medium-Range Weather Forecasts (ECMWF) Re-Analysis (ERA) Interim dataset. The SST data were obtained from the TRMM Microwave Imager (TMI) dataset, provided by the Remote Sensing System (RSS).
